# Factors associated with work ability among employees of an Italian university hospital

**DOI:** 10.1186/s12913-023-10465-z

**Published:** 2024-01-04

**Authors:** Loretta Casolari, Ylenia Curzi, Michele Mastroberardino, Barbara Pistoresi, Erica Poma, Lorenzo Broccoli, Tommaso Fabbri

**Affiliations:** 1 University Hospital of Modena, Modena, Italy; 2 University of Modena and Reggio Emilia, Modena, Italy

## Abstract

**Background:**

A growing body of evidence clearly documents the benefits of integrated systems approaches to protecting and promoting the safety, health and well-being of workers. The purpose of this study is to provide a holistic view of the work ability of employees of an Italian University Hospital measuring their resources in relation to job demands. In particular, it examines socio-demographics, family and organizational antecedents of health professionals’ work ability.

**Methods:**

A survey was conducted to assess the work ability of healthcare professionals, including physicians, nurses and administrative staff, working at the University Hospital of Modena (Italy). The data collection allows us to get a sample of 443 workers, who correspond to 11% of the target population. The data were analyzed using preliminary statistics on the main characteristics of the sample in terms of work ability, socio-demographic variables, family and organizational characteristics. In addition, logit models of the likelihood of having high work ability were estimated using SPSS version 25.

**Results:**

Work ability decreases with increasing age, comorbidity, high body mass index, having at least one child under 5 and/or a dependent adult, having a poor work-life balance, and doing more than 20 h of housework. Specific job resources can significantly promote work ability, including relationship-oriented leadership, autonomy in decision making and individuals’ skill match. The nursing profession is associated with a low work ability. Finally, a significant gender gap has been documented. Women find it more difficult to reconcile life and work, especially when they have children of preschool age and work in professions with greater responsibilities, as in the case of women doctors, who experience lower work capacity.

**Conclusions:**

Our results suggest that it is necessary to consider other factors, in addition to age, that are equally relevant in influencing work ability. Consequently, organisational interventions could be implemented to improve the work ability of all workers. In addition, we propose targeted interventions for groups at risk of reduced work capacity, in particular older workers (45 years and over), nurses, women with children of preschool age and in the position of physician.

**Supplementary Information:**

The online version contains supplementary material available at 10.1186/s12913-023-10465-z.

## Background

The aging of the working population has prompted governments in many industrialized countries to take measures to raise the official retirement age in order to avoid the collapse of pension systems and to address labor shortages [1]. It is estimated that by 2050, one in three people in developed countries will be 60 years old or older [1].

This trend is particularly critical in those sectors characterized by significant budget cuts, such as the public health sector, where increased demand for public health services, in the face of workforce shortages and a slowdown in new recruitment may lead to an increased incidence of burnout, early retirement, increased occupational illness and injury, as well as lower quality of services provided [2–4]. The relevance of this topic has also been highlighted by the pandemic emergency, which has also had serious consequences and impacts on the well-being of health workers, making the design and evaluation of indicators of workers’ health and work capacity particularly important.

In order to maintain the health of health professionals, hospitals have started to pay attention to the monitoring of the work ability of employees, a concept first developed by the Finnish Institute of Occupational Health in the early 1990s, which refers to the physical and mental capabilities of individuals in relation to their job role, implying a balance between personal resources and work demands [5, 6]. Specifically, the most widely adopted and best known indicator for measuring work ability is the work ability index [5, 7].

Empirical studies on the work ability show that this is a relevant predictor of early retirement, sickness absence and intention to leave the organization [8–10]. Furthermore, good levels of work ability are associated with a safer workplace, improved performance, a better quality of life and workers’ well-being [11–13]. Ruitenburg et al. [4], focusing on hospital physicians, shows that burnout and work-related stress are associated with lower work ability. In turn, low work ability, the presence of psychological distress or mental illness could lead to reduced performance, increased risk of long-term sick leave, and increased patient safety risk associated with increased likelihood of errors [14, 15]. Therefore, work ability can also be used as a preventive tool to examine the health status of employees by identifying potential future health problems and declines in work performance.

In summary, among the indicators related to the quality of ageing, the literature agrees that work ability is a good indicator to assess the health status of workers and their current ability to continue working [6, 9].

Despite its important role as a predictor, most studies focus on the determinants of this dimension, finding significant associations of it with the biological, physical, and psychological characteristics of individuals. Specifically, lower levels of work ability are associated with increasing age, job tenure smoking habits, obesity status, alcohol abuse, the presence of comorbidities and musculoskeletal disorders [16–20].

Recently, human resource management has paid increasing attention to organisational characteristics, although empirical evidence in this area is still in its infancy [7, 21]. In particular, some study have found that shift work and work pressure reduce the work ability for certain groups of workers [22, 23], while organizational resources, such as supervisor support, autonomy, discretion and team work often lead to an improvement in the work ability [12, 24–27].

Finally, little attention has been paid to the ‘external factors’ that influence work ability, such as work-family conflict, high household workload and their correlation with gender differences [28, 29]. Some studies find that women have lower work ability than men under certain conditions, but few studies explore this aspect between different work, family and social contexts [16, 18, 22, 30].

The contribution of this study is twofold. First, we provide a holistic and comprehensive picture of the main determinants of the work ability, taking into account socio-demographic, family and organizational antecedents of work ability of health professionals, through the adoption of the Job Demands-Resources Model and the integrated perspective provided by the Work Ability House. Second, we delve into the presence of gender differences between specific job roles and family characteristics (e.g., having at least one child under the age of 5), which have received little attention until now. In this respect, our work highlights the need to consider factors other than aging that are equally important in influencing work ability, and therefore envisages a wide range of interventions at different levels, thus responding to the call for more research in this area of analysis [31].

## Methods

### Study design and sampling

A survey for the data collection of this study was conducted at the University Hospital of Modena, the chief town of an Italian province of about 700,000 inhabitants in the Emilia-Romagna region of northern Italy, to assess the work capacity of health professionals and its association with other socio-demographic and organizational characteristics. The hospital has approximately 4200 employees including physicians, nurses and administrative staff.

According to the current Italian occupational health and safety legislation, University Hospital workers exposed to occupational risks must undergo a regular Health Surveillance (HS) program implemented by the Occupational Health Surveillance service (OHS). All University Hospital workers involved in the HS program were considered potentially eligible for the study. The inclusion criteria were to be a worker of the University Hospital and to be at medical examination between the 1 August 2022 and the 30 September 2022. No age restrictions or other exclusion criteria have been applied. The study was conducted according to the guidelines of the Declaration of Helsinki, and approved by the Ethics Committee of the “Area Vasta Emilia Nord” (reference number 185/2022/SPER/AOUMO SIRER ID 4136, protocol 0017051/22). Informed consent was obtained from all the available subjects involved in the study.

The data collection procedures ultimately allowed us to obtain a sample of 443 workers (physicians, nurses and administrative staff) representing 11% of the target population. With this sample, a logistic analysis of the probability of having a high work ability will be estimated.

Given the way the data were collected (physical presence of participants in the hospital between survey months and voluntary adherence) we obtained a convenience sample. Nevertheless, there is a fair degree of “similarity” between our sample and the target population with respect to some of the key characteristics (age, gender, job role, hospital areas). A detailed comparison between the sample and the target population is presented in Table 1.

**Table 1:** Descriptive statistics and comparison between sample and target population

	Sample		Population	
	N	%	N	%
**Total**	443	11%	4217	100%
**Gender**				
F	363	82%	3118	74%
M	80	18%	1099	26%
**Age group**				
<35	86	19%	894	21%
35–44	84	19%	1047	25%
45–54	129	29%	1163	28%
>54	144	33%	1113	26%
**Job role**				
Physicians	78	18%	824	20%
Nurses	290	65%	2923	69%
Administration	75	17%	470	11%

### The questionnaire

The questionnaire was structured into three sections: a first section in which respondents indicate their socio-demographic information such as their age, gender, characteristics of the household, amount of domestic work, quality of work-life balance. The second part of the survey includes the Work Ability Index (WAI). There has been a rapid proliferation of different indicators that can measure the work ability of employees, but in this study we use the most widely accepted and well-known indicator [5, 7], the Work Ability Index. Alternative indicators are usually based only on perceived work ability, such as the Work Ability Score and Perceived Work Ability, whereas the Work Ability Index allows us to include both objective characteristics and subjective perceptions [32]. In this regard, there are studies that point out some criticisms of this indicator, including its constructive characteristics, i.e. the inclusion of several factors within the same index. Specifically, according to some studies, perceived characteristics and “objective” data should be kept in separate items [12, 32]. Despite this, we believe that the Work Ability Index is still the preferred option of the majority of studies, as the inclusion of illnesses and medical indicators also provides a more complete picture of work ability, beyond just workers’ perceptions.

The Work Ability Index is a self-assessment comprising seven dimensions; the current ability to work compared to one’s lifetime best (0–10 points), one’s ability to work in relation to the physical and mental demands of the job (1–5 points), number of current illnesses diagnosed by a physician (list of 51 illnesses), estimated reduction in ability to work due to diseases (1–6 points), number of days of sick leave in the past 12 months, one’s prognosis of expected ability to work in two years (1–3 points), personal resources (1–5 points). A detailed description of the work ability index and variables in this indicator is provided in Table A of the Appendix.

Although these questions are self-reported by the respondent, this indicator includes both subjective perceptions of work ability (e.g., question 1 “personal assessment of current work ability compared to lifetime best, question 2 “assessment of mental and physical demands of the job”) and characteristics that can be interpreted as objective (e.g., question 3 “number of illnesses diagnosed by a doctor,” question 4 “number of days of sick leave in the past 12 months”).

In fact, the WAI is a composite indicator that includes subjective and objective characteristics of individuals’ work ability. Its final score ranges from 7 to 49 points, usually this score can be grouped into 4 dimensions: low work ability (7–27), moderate (28–36), good (37–43), excellent (44–49), or two dimensions: low work ability (7–36) and good work ability (37–49).

Finally, the third section of the questionnaire includes organizational characteristics of the unit in which the worker is employed. This section comprises different work characteristics including the presence of shift work, the degree of autonomy and discretion, the degree of support of the supervisor and colleagues, and the perception of receiving adequate training.

The analysis of the variables was derived from the questionnaire provided in the Supplementary file, Table C. Note that the working conditions section was developed inspired by the European Working Conditions Survey (2017) [33], while the Work Ability Index was constructed using those proposed in Tuomi K., Ilmarinen J., Jankola A., Katajarinne L., Tulkki A. Work Ability Index, Finnish Institute of Occupational Health (1998) [6].

## Methodology

The data analysis of this article was performed using SPSS, v. 25. First, we present preliminary statistics on the main characteristics of the sample, such as low and high work ability, gender, age group, and family composition. We also compare these preliminary statistics between the men and women groups. Second, logit models on the likelihood of having a high work ability have been estimated.

### Variables

#### Work ability

The outcome variable adopted for this study is the WAI, which, as mentioned earlier, measures the work ability of individuals and ranges from a score of 7 to 49. We adopt this variable in its dichotomized scale. Specifically, we create a dummy variable = 1 when work ability is high (37–49) and = 0 if work ability is low (7–36).

### Job roles

The job roles were classified into three categories: physicians, nurses and nursing aides, administrative staff. To synthesize, we will abbreviate the category nurses and nursing aides in nurses, and the category administrative staff in administrative.

#### Socio-demographic and family characteristics

The variables related to socio-demographic characteristics include age, which was grouped into four categories (< 35 years, i.e. 23–34; 35–45; 45–55 ;> 55 years), the dummies relating the worker gender, equal to 1 for women, having at least one child under the age of 5 years, having a adult dependent, doing housework (e.g. cooking, cleaning etc.) for more than 20 h per week. A work-life balance variable measured in likert scale (1–5, where low levels identify a scarce work-life balance and high levels identify a good work-life balance). Having a BMI associated with obesity, comorbidities (having more than three diseases together).

#### Organizational characteristics

According to the JD-R model [34] organizational characteristics can be divided among job demands and job resources, we analyse the following variables, classified in these two categories:

1. Job Demands.
  *Shift work*: dummy variable = 1 if the respondent performs shift work, = 0 otherwise.
2. Job Resources.

- *Supervisor support*: dummy variable = 1 for a good level of support and = 0 when the support is scarce or null.
- *Relationship-oriented leadership*: dummy variable = 1 if the supervisor adequately supports the professional growth and development of the employee, = 0 otherwise.
- *Colleagues support*: dummy variable = 1 for a good level of support and = 0 when the support is scarce or null.
- *Skill match*: dummy variable = 1 if the worker believes to have adequate competencies in relation to the job requirements, = 0 otherwise.
- *Autonomy over departmental or corporate organizational choices*: dummy variable = 1 when the worker participates in the improvement of the work processes and of the organization, = 0 if otherwise.
- *Autonomy over work goals*: dummy = 1 if the worker has autonomy in establishing the work objectives, = 0 otherwise.
- *Autonomy in decision-making*, dummy = 1 if the worker has autonomy in influencing the decisions that are important for his/her work, = 0 otherwise.

## Results

### Descriptive statistics

As previously mentioned, our sample represents around 11% of the target population, with a good degree of similarity between some key characteristics. In particular we find a good level of similarity in terms of gender, age, distribution across hospital areas and job roles. Table 1 summarizes these comparisons. In both the population and the sample, the majority of workers are women, the distribution among the different age groups is fairly even, while in terms of hospital area, the medical ward area comprises the majority of health care workers in both the sample and the whole population. With respect to job roles, most of the workers belong to the nurse category in the sample as well as in the whole population.

According to Table 2, a substantial portion of the sample appear to be in poor health: 35% have a low work ability and 43% have three or more illnesses. The presence of a significant proportion of workers in poor health may be related to the higher concentration of employees among the nursing category, which is typically characterized by a lower health status due to the considerable physical and caring efforts required in normal work activities. In addition, a portion of the sample has family conditions that may not facilitate work-life balance. Indeed, 30% of the sample report having a poor work-life balance, a quarter of the sample has at least one dependent adult, 22% have at least one child under the age of 5, and more than half of the sample (51%) declares that they spend more than 20 h a week on housework, including cooking and cleaning.

**Table 2:** Descriptive statistics, work ability and socio-demographic characteristics

Variables	Total		Women		Men	
	%	(N)	%	(N)	%	(N)
**Work ability**						
High WAI	65%	290	63%	230	75%	60
Low WAI	35%	153	37%	133	25%	20
**Age group**						
< 35	19%	86	17%	62	30%	24
35–44	19%	84	18%	65	24%	19
45–54	29%	129	31%	113	20%	16
> 54	33%	144	34%	123	26%	21
**Job role**						
Physicians	18%	78	16%	59	24%	19
Nurses	65%	290	67%	241	50%	40
Administratives	17%	75	17%	63	26%	21
**Health conditions**						
Workers with no diseases	27%	120	26%	94	32%	26
1–2 diseases	30%	131	28%	100	39%	31
3 or more diseases	43%	192	46%	169	29%	23
Obesity	14%	62	14%	50	15%	12
**Household and work-life balance**						
Dependent adults	25%	109	26%	91	23%	18
Children less than 5 years old	22%	98	21%	77	26%	21
Scarce work-life balance	30%	132	30%	107	31%	25
Houseworks	51%	227	53%	194	41%	33

Note that the incidence of workers with a low WAI is higher for women, where 37% have a low WAI, compared to 25% of men, and, in addition, 47% of women have three or more conditions, while this percentage is 18% lower in men, where 29% report having three or more conditions. The worse health status of women may be associated with the greater amount of hours of housework they declare doing: in fact, while there is no significant difference between the percentage of women and men with dependent adults or children under 5 years of age, the percentage of women doing housework for more than 20 h is particularly higher at 53%, while this value is reduced to only 41% of men. Other reasons that may explain the lower levels of work ability in women may be associated with the higher incidence of nurses among women (66%), compared to men (51%), or the higher concentration of women among the over-54s (34% of women and 26% of men).

Table 3 reports logistic regressions on the probability of having a high WAI with respect to sociodemographic characteristics, health status, family composition, and working conditions (Models 1–5). In addition, some interactions are presented to assess the presence of gender differences in relation to work-life balance issues (Models 2–5). In our case, the final sample that we use in the logit models covers N = 354 because of missing values (non-responses) resulting in the exclusion of 20% of the respondents, although the missing values for each variable do not exceed 17%. Variables in which the percentage of missing is slightly higher are *Skill match* (15%), *Children less than 5 years old* (11%) and *Housework* (17%). This smaller sample size does not appear to increase the standard errors of the variables of interest by reducing their significance level.

**Table 3:** Logistic regression models of the probability of having a high WAI

Variables	(1)	(2)	(3)	(4)	(5)
	Model 1	Model 2	Model 3	Model 4	Model 5
**Age and gender**					
Under 35 age group	2.099***	2.168***	2.209***	2.101***	2.148***
	(0.292)	(0.258)	(0.236)	(0.299)	(0.399)
35–44 age group	1.240***	1.196***	1.208***	1.233***	1.285***
	(0.262)	(0.315)	(0.211)	(0.286)	(0.305)
45–54 age group	0.682	0.680	0.730	0.681	0.667
	(0.574)	(0.551)	(0.541)	(0.574)	(0.612)
Female	0.621	1.106***	-3.121	0.754	0.693
	(0.563)	(0.274)	(1.949)	(0.561)	(0.564)
**Job role**					
Nurses	-0.554***	-0.647***	-0.584***	-0.572***	-0.577***
	(0.0462)	(0.154)	(0.0751)	(0.0457)	(0.0359)
Physicians	0.186	2.056***	0.0671	0.139	0.158
	(0.142)	(0.325)	(0.248)	(0.128)	(0.120)
**Health conditions**					
Diseases	-0.597***	-0.610***	-0.608***	-0.598***	-0.625***
	(0.0483)	(0.0351)	(0.0482)	(0.0440)	(0.0519)
Obesity	-0.722**	-0.820***	-0.810***	-0.713***	-0.677***
	(0.286)	(0.203)	(0.135)	(0.275)	(0.185)
**Family conditions and work-life balance**					
Dependent adults	-0.622***	-0.657***	-0.662***	-0.619***	-0.515***
	(0.0514)	(0.0356)	(0.0873)	(0.0422)	(0.0853)
Children	-0.170	-0.274***	-0.169	0.248	4.616***
	(0.122)	(0.010)	(0.116)	(0.213)	(0.918)
Work-life balance	0.609***	0.634***	-0.219	0.605***	0.988***
	(0.190)	(0.175)	(0.574)	(0.188)	(0.214)
Housework	-0.571***	-0.593***	-0.588***	-0.592***	-0.628***
	(0.119)	(0.129)	(0.143)	(0.115)	(0.131)
**Job resources and demands**					
Shiftwork	-0.730***	-0.693***	-0.724***	-0.742***	-0.725***
	(0.138)	(0.173)	(0.171)	(0.132)	(0.159)
Relationship-oriented leadership	0.293***	0.282**	0.283***	0.291***	0.332***
	(0.102)	(0.113)	(0.0946)	(0.102)	(0.0744)
Colleagues support	0.0160	-0.00203	0.0206	0.00696	-0.0114
	(0.204)	(0.189)	(0.190)	(0.199)	(0.202)
Autonomy over work goals	-0.262	-0.244	-0.271*	-0.246	-0.221
	(0.179)	(0.190)	(0.160)	(0.186)	(0.168)
Autonomy in decision making	0.300***	0.282***	0.311***	0.295***	0.277***
	(0.083)	(0.072)	(0.088)	(0.091)	(0.041)
Autonomy in departmental choices	0.211	0.225	0.209	0.213	0.172
	(0.152)	(0.141)	(0.158)	(0.154)	(0.142)
Skill match	1.933**	1.866**	1.942**	1.924**	1.981***
	(0.877)	(0.944)	(0.867)	(0.859)	(0.594)
**Interactions**					
Fem*physicians		-2.542***			
		(0.279)			
Fem*work-life balance			1.035**		
			(0.453)		
Fem*Children				-0.524***	
				(0.188)	
Work-life balance*Children					-1.313***
					(0.230)
Constant	-5.400***	-5.534**	-2.333	-5.439***	-6.819***
	(1.923)	(2.299)	(3.064)	(1.894)	(1.570)
Pseudo R^2^	0,415	0,427	0,424	0,416	0,417
Observations	354	354	354	354	354

Robust standard errors in parentheses, clustered for job title. *** p < 0.01, ** p < 0.05, * p < 0.1

### Socio-demographic characteristics, health conditions and job role

Table 3 shows that the probability of having a high WAI decreases with age; in particular, only the under-35 and 35–44 age groups have a significantly higher average WAI than the over-55 age group, while the 45–54 age group is not significantly different from the over-55 age group. This result is robust to all logistic specifications from Model 1 to Model 5, Table 3. Note that because of the high correlation, we only consider the relationship between WAI and age groups, rather than the relationship with job tenure, as these two variables are highly correlated.

In addition, we will detail below how gender differences seem to emerge in specific job roles, captured by interactions (Models 2–4, Table 3), while in the overall sample the female dummy alone does not show significant correlations with the WAI in most cases.

In terms of job role, our results show that nurses appear to be the occupational category with the lowest levels of WAI, in particular they have significantly lower levels than the excluded groups, i.e. *administrative staff*, and *physicians*.

The lower levels of WAI attributed to nurses are not surprising, as several works in the literature note that nurses and nursing assistants, given the nature of the work, that requires increased physical demands and stressful conditions, lead to a reduction in their well-being, linked to issues such as increased presence of musculoskeletal disorders and other related conditions [19, 22, 29, 35].

Regarding the health status of individuals, the presence of one or more diseases and a BMI associated with obesity, significantly reduce the likelihood of having a high WAI (Models 1–5, Table 3). These factors are frequently associated as important predictors of work ability, as they contribute to employees’ personal resources and job performance. This highlights the importance of promoting the adoption of healthy lifestyles and frequent sports activities even within organizations [36–38].

### Household composition and work-life balance

Particular attention is paid to dimensions related to household composition (i.e., having at least one child under the age of 5 and having an adult dependent), work-life balance, and the amount of domestic work of employees (specifically, performing more than 20 h of domestic work per week such as cooking, cleaning, ironing). In fact, according to the “WAI household” model, family and the surrounding social environment are important factors that, although external to organizations, have a major influence on WAI [28, 29, 39]. Our results confirm their decisive influence for most of these predictors in Table 3. Specifically, having a dependent adult significantly and negatively reduces the likelihood of having a high WAI across all the specifications. Differently, having a child is significantly and negatively associated with a high work ability only in Model 2 and 5, while in the other cases it registers a negative but non-significant association. In other words, it appears that having an adult dependent has a greater influence on individuals’ ability to work than having a young child. However, we will investigate the effect of the latter variable in interactions with the female dummy subsequently. Also, the work-life balance is an important factor, since a good level of balance significantly increases the likelihood of having a high WAI (Models 1, 2,4,5, Table 3). On the other hand, having an amount of housework that requires more than 20 h of work per week significantly reduces the likelihood of having a good level of WAI (Models 1–5, Table 3).

### Organisational characteristics

Turning to the organizational antecedents that influence the work ability, our results show that, firstly, shift work is significantly associated with a reduction in the probability of having a high WAI. In fact, shift work and night work have been shown to be risk factors for several psychosomatic disorders as they can affect psychophysical homeostasis, efficiency, and social relationships [22].

On the other hand, a significant element that improves the likelihood of having high work ability is the presence of relationship-oriented leadership. In particular, a supervisor who encourages the worker’s professional development seems to be a relevant characteristic. The relevance of one’s supervisor’s support was also found in other work settings [32, 40, 41]. Our results confirm that the likelihood of having high work ability is not determined by “general’’ daily support, but, rather, by specific relationship-oriented leadership support that promotes employees’ professional careers (See also Model 1, Table B in the Appendix).

Autonomy is an important resource for promoting a higher work ability [41]. In particular, we find that having autonomy in decision-making significantly and positively increases the likelihood of having a high WAI.

Finally, the *skill match*, which identifies an individual’s perception of having adequate skills in relation to job requirements, is significant in increasing the likelihood of having a high WAI. Previous studies on work ability have not paid particular attention to this organizational characteristic, however, the positive and significant sign for all the specifications in Table 3 leads us to argue that this is an important dimension to consider.

### Interactions

Although the dichotomous variable *Female* shows in most cases no significant association with WAI, we find that the interaction Fem*physician turns out to be statistically significant and negatively associated with a high WAI. In other words, while in the total sample there are no differences in terms of WAI between women and men, we find that among physicians, women are significantly less likely than other professional categories and male physicians to have a high WAI (Model 2, Table 3). We also evaluate the presence of gender differences between nurses and administrative positions, finding no significant effect among nurses, while in the administrative category women are more likely to have a high WAI (Models 2–3, Table B, in the Appendix).

These gender differences could be explained by the greater work-life balance difficulties for physicians, especially in cases where the domestic workload is high (e.g., if one has young children), while in the case of administrative staff, work-life balance problems may be less challenging due to different working hours and the absence of the night shift.

In fact, the category of physicians is characterized by increased shift work and long hours (e.g., more than 10 consecutive hours). Since women may be unequally subjected to a greater domestic workload, they may have greater difficulties in achieving work-life balance in this particular profession. It is important to note that the greatest work-life balance difficulties among physicians are also found in other studies, such as that of La Torre et al. [29], which found that work-family conflict is higher for women and physicians, while administrative staff is the category with the lowest levels of work-family conflict; in addition, Treister-Goltzman [42] and Adam et al. [43] found that women physicians are subject to greater difficulties in work-family conflict.

Furthermore, the interaction fem*work-life balance shows a significant and positive effect on WAI, indicating that for women, a good work-life balance has a greater effect in increasing the likelihood of having a high WAI (Model 2, Table 3). In addition, the interaction female*children shows a significant and negative association with WAI, suggesting that women with at least one child under age 5 have a lower probability of having a high WAI than all other workers and men with children under age 5 (Model 3, Table 3).

These effects suggest that for women, work-life balance is more significant in influencing WAI and, in addition, having at least one young child significantly reduces the probability of having a high WAI. Finally, the interaction Work-life balance*children is negative and significant, supporting that having a young child significantly reduces the positive effect of having a greater work-life balance on the probability of having a high WAI, for all genders (Model 5, Table 3).

This result can be attributed to the greater difficulty for women to balance work and private life against an unequal amount of housework. We see that women with small children have lower levels of WAI; furthermore, having small children significantly reduces the positive effect of work-life balance on WAI; as the medical profession is particularly prone to long shifts and intense workloads, work-life balance may be penalised with a worse effect for women.

## Discussion

These results shed light on a number of determinants of work ability that cover different levels of analysis, including health and demographic dimensions of the person, work and organisational characteristics, and characteristics related to the family and social sphere.

In relation to individuals’ socio-demographic and health characteristics, we find that the likelihood of having a high WAI decreases at increasing age, presence of comorbidities and when the workers have an obesity status. These results are sharply confirmed by previous empirical studies [16, 18–20, 30]. Furthermore, the roles of nurses and nurse aides are characterised by a lower likelihood of high work ability. In connection with this, many studies also agree that work ability depends on the type of activity performed, thus determining why certain occupations, that require a great deal of physical effort, are characterised by a more rapid decline in work ability, such as the cases of nurses, midwives and manual workers [19, 20, 29]. For instance, the profession of nurses and nurse aides could also be subject to a great incidence of specific pathologies, such as musculoskeletal disorders or chronic forms of disability, that may also contribute to deteriorate work ability [19, 22, 36, 44].

Beyond socio-demographic characteristics, recent works agree that a more comprehensive analysis of the determinants of work ability is requested, thus proposing the conceptual model of the Work Ability House, which allows for an integrated analytical framework to understand the determinants of work ability, including household composition and social community [39]. In this regard, we find that having a good work-life balance, having at least one adult dependent, and doing more than 20 h of domestic work per week are important predictors of WAI, as the sign and significance of these variables are robust across all model specifications. It should be noted that the important influence of work-life balance dynamics has only been emphasized in a few recent articles, highlighting the importance of further analysis of these dimensions [28, 29, 41].

For instance, Smyth, Pit and Hansen [28] find that the role of the family and social community is crucial in determining work ability and that there are significant gender impacts, with women having lower levels of work ability depending on their work-life balance. We find different results which confirm this idea. In fact, our results confirm that for women work-life balance has a greater effect on work ability than male and, moreover, we also find that women with young children are less likely to have high work ability than men in the same situation. Furthermore, women that are physicians have a lower probability of having a high WAI than men physicians and this can be also due to the greater difficulties in balancing work and life spheres for this type of job occupation, characterized by longer shift hours and responsibilities. In relation to this, La Torre et al. [29] found that for female physicians the work-family conflict is higher.

With regard to disadvantages that may arise from the social environment of individuals, the literature on this topic is still in its infancy, but there is a consensus on the need to find instruments of social support. This may refer to social partners, collective agreements and national policies which could address this issue reducing social inequalities, with positive effects on the promotion of work ability for the vulnerable groups of the population [39]. Social support should also be provided by employers through benefits such as health insurance, childcare benefits, financial bonuses and paid time off, as well as by supervisors and colleagues [41].

Specifically, organizational studies on work ability often adopt the Job Demands-Resources (JD-R) model to differentiate between job demands, such as shift work, working for long hours, emotional exhaustion, and job resources, such as supervisor and c-worker support, autonomy and discretion levers, flexible work schedule [24, 25, 34].

We find that relationship-oriented leadership, autonomy in decision-making and skill match are important resources which can help in promoting work ability, as confirmed by previous works [32, 40, 41]. On the other hands, these resources can help in preventing the early deterioration of work ability resulting from the negative effect of job demands, such as shift works, which, in our study, result to significantly decrease the likelihood of having a high work ability, confirming results of previous works [22]. Organisational resources are, therefore, key elements as they are also important psychosocial factors that, according to other works, can improve the working ability of individuals, especially for the most vulnerable groups [45, 46].

## Conclusion and practical implications

Sustainable employability, that is the ability to function adequately at work throughout working life, is a key aspect in healthcare as it deals with mismatches between job resources and working environment, and it supports the retention of trained specialists who are difficult to replace because of their expertise [47, 48]. High work ability in hospitals means, not only greater wellbeing for healthcare professionals, but also lower costs for the organization and better services for patients, with positive spillover effects for the wider community.

The existing literature on the WAI emphasizes the positive association between work ability and age of the workforce. Our work also highlights the need to consider factors other than aging that are equally important in influencing work ability, and therefore envisages a wide range of interventions at different levels, thus responding to the call for more research in this area of analysis [31]. Although there is limited evidence supporting a favorable effect of workplace interventions to improve work ability [21, 49], it is important to better identify which aspects of interventions are effective in achieving this goal within the healthcare system.

At the individual level, personal resources may be enhanced by individuals adopting healthy lifestyles, which allow them to reduce the likelihood of suffering from illness and can help maintain a BMI associated with normal weight. Although these elements are based on personal choices, organizations can offer psychological support and counseling activities, to promote the workers’ overall mental and physical well-being and their attitude toward a healthier lifestyle. Particular attention could be paid to nurses and allied health professionals (such as nursing assistants and other non-physician health workers), who perform physically demanding tasks and have significantly lower WAI values than other health professionals. This will require the provision of appropriate equipment, but also special programmes and agreements with professionals (e.g. physiotherapists).

It is important to address all factors when promoting a change in behaviour. Promoting a change in physical activity or dietary behaviour, for example, should include changes in the workplace’s physical environment and organizational structure (availability of healthy foods, policies, etc…) [50]. At the organizational level, interventions should also promote relationship-oriented leadership, that is, supervisors should pay attention to supporting employees in their personal and professional development. Importantly, organizations may promote human resource development practices such as on-the-job training, training courses, and similar activities that enhance individual competencies, as these elements increase the individual’s skill match and the likelihood of high work ability [51]. In this regard, a relationship-oriented leadership is a key variable, and therefore the supervisor should be able to notice and support more individuals who may be suffering from a skills mismatch [10]. Furthermore, giving employees more influence over decision-making processes can help promote their work ability. On the other hand, shift work should, when possible, be limited, given its negative effect on the work ability. The above-mentioned interventions may be particularly useful for the over-45s, who are less likely to have a high level of work ability.

Given the relevant role of work-life balance and family composition in influencing the ability to work, hospitals could design services and care facilities for workers with dependent adults. In a similar way, hospitals could design policies for in-house kindergartens for their workers; we believe that this important practice should be strengthened and promoted as it can improve the work-life balance of individuals, especially women, thereby increasing their ability to work.

Our study points to strong gender differences in the medical profession encouraging future study to explore this issue in more depth, especially in relation to domestic work (caring, cooking, cleaning) and work-life balance. In summary, we believe that future studies on work ability should try to focus more on the determinants related to the social and family environment of employees, deepening the understanding of how household characteristics, family duties and the social environment close to individuals, including friends, relatives and the community, may influence work ability. Furthermore, future studies should better address the relationship between social inequalities and early deterioration of working ability. In particular, family and social determinants could be a disadvantage, rather than a source of support, in influencing work ability, especially for vulnerable population groups. Finally, future studies should seek to investigate the effects of organisational interventions, such as on-the-job training, supervisor mentoring, coaching, and the effects of relationship-oriented leadership, as intervention-based studies are few, especially with regard to organisational interventions, and more attention should be paid to this topic [31, 49].

This study also has some major limitations. Firstly, our results are based on a convenience sample of participants who voluntarily and anonymously took part in the survey, which allowed us only to reach percent of the reference population. This non-probability sample is not selected to be statistically representative of the reference population, so there is no direct relationship between the size of our sample and the size of the target population. Furthermore, although our sample is similar to the reference population in terms of some occupational and demographic characteristics, it may not accurately reflect all characteristics of the population. Therefore, our future efforts will be directed towards constructing a probabilistic sample from which we can obtain results that can be generalised to the whole population.

Secondly, our logistic model estimates were obtained on a small number of observations due to the presence of missing values, approximately 20%. A proportion of the respondents did not complete the survey and for this reason some observations were eliminated in the estimation of the logistic models.

The data of this study may be relevant for the further development of health and policy strategies in the workplace and that would be described as TWH programs by the NIOSH definition (http://www.cdc.gov/niosh/twh/totalhealth.html ).

### Electronic supplementary material

Below is the link to the electronic supplementary material.

Supplementary Material 1

## Data Availability

The datasets used and/or analysed during the current study available from the corresponding author on reasonable request.

## References

[1] United Nations, Department of Economic and Social Affairs. World Population Prospects: The 2017 Revision, Key Findings and Advance Tables. Published online 2017.

[2] Cantarelli P, Vainieri M, Seghieri C (2023). The management of healthcare employees’ job satisfaction: optimization analyses from a series of large-scale surveys. BMC Health Serv Res.

[3] OECD. Engaging public employees for a high-performing civil service. OECD; 2016. 10.1787/9789264267190-en.

[4] Ruitenburg MM, Frings-Dresen MH, Sluiter JK (2012). The prevalence of common mental disorders among hospital physicians and their association with self-reported work ability: a cross-sectional study. BMC Health Serv Res.

[5] Ilmarinen J, Tuomi K (1992). Work ability of Ageing workers. Scand J Work Environ Health.

[6] Tuomi K, Ilmarinen J, Jahkola A, Katajarinne L, Tulkki A. Work ability index. Volume 19. Finnish Institute of Occupational Health Helsinki; 1998.

[7] van den Berg TIJ, Elders LAM, de Zwart BCH, Burdorf A (2008). The effects of work-related and individual factors on the work ability index: a systematic review. Occup Environ Med.

[8] Tisch A (2015). Health, work ability and work motivation: determinants of labour market exit among German employees born in 1959 and 1965. J Labour Market Res.

[9] Pak K, Kooij DTAM, De Lange AH, Van Veldhoven MJPM (2019). Human Resource Management and the ability, motivation and opportunity to continue working: a review of quantitative studies. Hum Resource Manage Rev.

[10] Kunz C, Millhoff C (2023). A longitudinal perspective on the interplay of job demands and destructive leadership on employees’ work ability in Germany. Int Arch Occup Environ Health.

[11] Backes-Gellner U, Veen S (2013). Positive effects of ageing and age diversity in innovative companies - large-scale empirical evidence on company productivity: Productivity effects of age-diverse workforces. Hum Resource Manage J.

[12] Brady GM, Truxillo DM, Cadiz DM, Rineer JR, Caughlin DE, Bodner T (2020). Opening the black box: examining the nomological network of work ability and its role in organizational research. J Appl Psychol.

[13] Rashid M, Heiden M, Nilsson A, Kristofferzon ML (2021). Do work ability and life satisfaction matter for return to work? Predictive ability of the work ability index and life satisfaction questionnaire among women with long-term musculoskeletal pain. BMC Public Health.

[14] Sell L (2009). Predicting long-term sickness absence and early retirement pension from self-reported work ability. Int Arch Occup Environ Health.

[15] Hilton MF, Whiteford HA (2010). Associations between psychological distress, workplace Accidents, workplace failures and workplace successes. Int Arch Occup Environ Health.

[16] El Fassi M, Bocquet V, Majery N, Lair ML, Couffignal S, Mairiaux P (2013). Work ability assessment in a worker population: comparison and determinants of work ability index and work ability score. BMC Public Health.

[17] Mokarami H, Cousins R, Kalteh HO (2022). Comparison of the work ability index and the work ability score for predicting health-related quality of life. Int Arch Occup Environ Health.

[18] Marzuca-Nassr GN, Soto-Rodríguez FJ, Bascour-Sandoval C (2021). Influence of age on functional capacity and work ability in Chilean workers: a cross-sectional study. Int Arch Occup Environ Health.

[19] Garzaro G, Clari M, Ciocan C (2022). Physical Health and work ability among Healthcare Workers. A cross-sectional study. Nurs Rep.

[20] Yi X, Yang J, Gao X, Li F (2022). The relationship between occupational stress, mental health and work ability of coal chemical workers in Xinjiang. Front Psychiatry.

[21] Oakman J, Neupane S, Proper KI, Kinsman N, NygÃ¥rd CH. Workplace interventions to improve work ability: a systematic review and meta-analysis of their effectiveness. Scand J Work Environ Health Published Online November. 2017;3. 10.5271/sjweh.3685. 10.5271/sjweh.3685 29493713

[22] Costa G, Sartori S (2007). Ageing, working hours and work ability. Ergonomics.

[23] Maurits EEM, De Veer AJE, Van Der Hoek LS, Francke AL (2015). Factors associated with the self-perceived ability of nursing staff to remain working until retirement: a questionnaire survey. BMC Health Serv Res.

[24] McGonagle AK, Barnes-Farrell JL, Di Milia L (2014). Demands, resources, and work ability: a cross-national examination of health care workers. Eur J Work Organizational Psychol.

[25] McGonagle AK, Fisher GG, Barnes-Farrell JL, Grosch JW (2015). Individual and work factors related to perceived work ability and labor force outcomes. J Appl Psychol.

[26] Converso D, Sottimano I, Guidetti G, Loera B, Cortini M, Viotti S (2018). Aging and work ability: the moderating role of Job and Personal resources. Front Psychol.

[27] Brunner B, Igic I, Keller AC, Wieser S (2019). Who gains the most from improving working conditions? Health-related absenteeism and presenteeism due to stress at work. Eur J Health Econ.

[28] Smyth J, Pit SW, Hansen V (2018). Can the work ability model provide a useful explanatory framework to understand sustainable employability amongst general practitioners: a qualitative study. Hum Resour Health.

[29] La Torre G, Grima D, Romano F, Polimeni A. Perceived work ability and work-family conflict in healthcare workers: an observational study in a teaching hospital in Italy. J Occup Health. 2021;63(1). 10.1002/1348-9585.12271. 10.1002/1348-9585.12271 PMC8448582 34535041

[30] Mokarami H, Kalteh HO, Marioryad H (2020). The effect of work-related and socio-demographic factors on work ability index (WAI) among Iranian workers. WOR.

[31] Söderbacka T, Nyholm L, Fagerström L (2020). Workplace interventions that support older employees’ health and work ability - a scoping review. BMC Health Serv Res.

[32] Cadiz DM, Brady G, Rineer JR, Truxillo DM. A Review and Synthesis of the Work Ability Literature. Wang M, ed. *Work, Aging and Retirement*. 2019;5(1):114–138. 10.1093/workar/way010.

[33] Parent-Thirion A, Biletta I, Cabrita J (2017). 6th European Working conditions Survey: Overview Report. 2017 update. Publications Office of the Euroepan Union.

[34] Demerouti E, Bakker AB, Nachreiner F, Schaufeli WB (2001). The job demands-resources model of burnout. J Appl Psychol.

[35] Marina Fischer F, Notarnicola Da Silva Borges F, Rotenberg L (2006). Work ability of Health Care Shift workers: what matters?. Chronobiol Int.

[36] Calatayud J, Morera Á, Ezzatvar Y (2022). Importance of frequency and intensity of strength training for work ability among physical therapists. Sci Rep.

[37] Rieker JA, Gajewski PD, Reales JM (2023). The impact of physical fitness, social life, and cognitive functions on work ability in middle-aged and older adults. Int Arch Occup Environ Health.

[38] Candio P, Mujica FP, Frew E (2023). Socio-economic accounting of inequalities in excess weight: a population-based analysis. BMC Public Health.

[39] Ilmarinen (2019). From work ability research to implementation. IJERPH.

[40] Arshadi N, Zare R. Leadership effectiveness, perceived organizational support and work ability: mediating role of job satisfaction. Published online 2016.

[41] McGonagle AK, Bardwell T, Flinchum J, Kavanagh K (2022). Perceived work ability: a constant comparative analysis of workers’ perspectives. Occup Health Sci.

[42] Treister-Goltzman Y, Peleg R. Female Physicians and the Work-Family Conflict. 2016;18. 27430080

[43] Adam S, Gyorffy Z, László K (2009). High prevalence of job dissatisfaction among female physicians: work-family conflict as a potential stressor. Orv Hetil.

[44] Amirmahani M, Hasheminejad N, Tahernejad S, Reza Tohidi Nik H (2022). Evaluation of work ability index and its association with job stress and musculoskeletal disorders among midwives during the Covid-19 pandemic. La Med Del Lavoro | Work Environ Health.

[45] Camerino D, Conway PM, Sartori S (2008). Factors affecting work ability in Day and Shift-Working nurses. Chronobiol Int.

[46] Lindberg P (2006). Promoting excellent work ability and preventing poor work ability: the same determinants? Results from the Swedish HAKuL study. Occup Environ Med.

[47] Fleuren BB, De Grip A, Jansen NW, Kant I, Zijlstra FR (2016). Critical reflections on the currently leading definition of sustainable employability. Scand J Work Environ Health.

[48] Roczniewska M, Richter A, Hasson H, Schwarz UVT (2020). Predicting Sustainable Employability in Swedish Healthcare: the complexity of Social Job resources. IJERPH.

[49] Cloostermans L, Bekkers MB, Uiters E, Proper KI (2015). The effectiveness of interventions for ageing workers on (early) retirement, work ability and productivity: a systematic review. Int Arch Occup Environ Health.

[50] Grimani A, Aboagye E, Kwak L (2019). The effectiveness of workplace nutrition and physical activity interventions in improving productivity, work performance and workability: a systematic review. BMC Public Health.

[51] Pak K, Kooij DTAM, De Lange AH, Heuvel S, Van Veldhoven MJPM (2021). The influence of human resource practices on perceived work ability and the preferred retirement age: a latent growth modelling approach. Hum Resour Manag J.

