# Supplementary material for: Factors associated with work ability among employees of an Italian university hospital
Source: BMC Health Serv Res. 2024 Jan 4;24:30. doi: 10.1186/s12913-023-10465-z (PMC10768426; doi:10.1186/s12913-023-10465-z)
Supplement: Supplementary file 1 — Supplementary Material 1 [file 12913_2023_10465_MOESM1_ESM.docx]

**SUPPLEMENTARY FILE**

**Table A**, **Work Ability Index, variables and dimensions**

| **Work Ability Index, dimensions** | **Number of Variables** |
| --- | --- |
| 1. The current ability to work compared to one's lifetime best | 1 Variable (*General*)  *“Assuming that your ability at its highest level has a value of 10, what score would you give to your current work ability?”*  From 0 to 10 points, where 0 = unable to work, 10= completely able to work |
| 1. One's ability to work in relation to the physical and mental demands of the job | 2 Variables (*Physical and Mental*)   - *What is your work ability in relation to the physical demands of the job?* - *What is your work ability in relation to the mental demands of the job?*   From 1 to 5 points, where 1 very bad, 5 very good |
| 1. Number of current illnesses diagnosed by a physician | 1 Variable ranged 0-51 depending on the number of diseases (*Diseases*)  *In the following list, please mark the diseases and/or injuries currently complained of by you; indicate whether a physician has diagnosed or treated such conditions.*  List of 51 specific illnesses, where 0 means no disease. |
| 1. Estimated reduction in ability to work due to diseases | 1 Variable *(Obstacles*)  *Does your health condition /illness obstruct your current work?*  From 1 to 6 points, where: 1= No obstacles, 6= Competely unable to work |
| 1. Number of days of sick leave in the past 12 months | 1 Variable (*Sick_leaves*)  *How many full days were you absent from work due to illness, treatment, examination, diagnostic tests in the last year (last 12 months)?*  From 1 to 5 points, where 1= No days, 2=less than 10 days; 3= from 10 to 24 days; 4= from 25 to 99 days, 5= more than 100 days |
| 1. One's prognosis of expected ability to work in two years | 1 Variables (*Future_WA*)  *Do you think that in relation to your current health condition you will be able to perform your current job in the next 2 years?*  From 1 to 3 points, where 1=Not very likely, 3= Very likely |
| 1. Personal resources | 3 Variables:  *In recent times have you been/are you able to perform your usual activities with satisfaction on a daily basis?*  *Have you felt active and alert in recent times?*  *Have you felt hopeful about the future in recent times?*  From 1 to 5 points, where 1=Never and 5=Always |

**Table B. Logistic regression models on the likelihood of having a high WAI**

| Variables | Model 1 | Model 2 | Model 3 | Model 4 | Model 5 |
| --- | --- | --- | --- | --- | --- |
|  |  |  |  |  |  |
| Under 35 age group | 2.130*** | 2.138*** | 2.032*** | 2.124*** | 2.138*** |
|  | (0.264) | (0.244) | (0.317) | (0.291) | (0.272) |
| 35-44 age group | 1.228*** | 1.248*** | 1.193*** | 1.227*** | 1.149*** |
|  | (0.278) | (0.261) | (0.310) | (0.270) | (0.305) |
| 45-54 age group | 0.658 | 0.662 | 0.723 | 0.683 | 0.642 |
|  | (0.540) | (0.573) | (0.527) | (0.573) | (0.558) |
| Female | 0.654 | 0.326 | 0.293 | 0.893 | 0.620 |
|  | (0.544) | (1.137) | (0.682) | (0.855) | (0.549) |
| Nurses | -0.572*** | -0.984 | -0.688*** | -0.741*** | -0.868*** |
|  | (0.0521) | (0.921) | (0.0871) | (0.122) | (0.168) |
| Physicians | 0.173 | 0.166 |  |  |  |
|  | (0.110) | (0.169) |  |  |  |
| Diseases | -0.602*** | -0.596*** | -0.607*** | -0.597*** | -0.596*** |
|  | (0.0489) | (0.0488) | (0.0365) | (0.0449) | (0.0414) |
| Obesity | -0.643*** | -0.754*** | -0.689** | -0.685*** | -0.830*** |
|  | (0.232) | (0.240) | (0.279) | (0.263) | (0.275) |
| Dependent adults | -0.633*** | -0.617*** | -0.663*** | -0.617*** | -0.665*** |
|  | (0.0711) | (0.0466) | (0.0662) | (0.0620) | (0.0831) |
| Children less than 5 years old | -0.190 | -0.178* | -0.211** | -0.211* | -0.193** |
|  | (0.144) | (0.104) | (0.0916) | (0.126) | (0.0912) |
| Work-life balance | 0.627*** | 0.616*** | 0.597*** | 0.610*** | 1.005*** |
|  | (0.176) | (0.178) | (0.175) | (0.184) | (0.315) |
| Housework | -0.594*** | -0.563*** | -0.596*** | -0.0423 | 1.977* |
|  | (0.183) | (0.114) | (0.107) | (0.770) | (1.147) |
| Shiftwork | -0.727*** | -0.717*** | -0.743*** | -0.739*** | -0.749*** |
|  | (0.138) | (0.164) | (0.156) | (0.141) | (0.145) |
| Relationship-oriented leadership |  | 0.287*** | 0.301*** | 0.289*** | 0.293*** |
|  |  | (0.107) | (0.0976) | (0.0943) | (0.0806) |
| General supervisor support | 0.266 |  |  |  |  |
|  | (0.250) |  |  |  |  |
| Colleagues support | -0.00319 | 0.0211 | -0.00422 | 0.000520 | 0.0604 |
|  | (0.308) | (0.207) | (0.178) | (0.190) | (0.209) |
| Autonomy over work goals | -0.225 | -0.264 | -0.248 | -0.254 | -0.233 |
|  | (0.158) | (0.175) | (0.193) | (0.174) | (0.189) |
| Autonomy in decision making | 0.317*** | 0.302*** | 0.277*** | 0.308*** | 0.344*** |
|  | (0.0876) | (0.0820) | (0.0804) | (0.0744) | (0.0746) |
| Autonomy in departmental choices | 0.219 | 0.212 | 0.219 | 0.199 | 0.151 |
|  | (0.168) | (0.146) | (0.150) | (0.142) | (0.165) |
| Skill match | 1.952** | 1.949** | 1.883** | 1.950** | 1.965*** |
|  | (0.849) | (0.916) | (0.863) | (0.866) | (0.732) |
| Fem*nurse |  | 0.537 |  |  |  |
|  |  | (1.098) |  |  |  |
| Administrative |  |  | -1.132*** | -0.166 | -0.326 |
|  |  |  | (0.384) | (0.116) | (0.206) |
| Fem*Administrative |  |  | 1.345** |  |  |
|  |  |  | (0.599) |  |  |
| Fem*Housework |  |  |  | -0.636 |  |
|  |  |  |  | (0.798) |  |
| Wlb*Housework |  |  |  |  | -0.697** |
|  |  |  |  |  | (0.288) |
| Constant | -5.524*** | -5.253** | -4.719*** | -5.397*** | -6.734*** |
|  | (1.847) | (2.088) | (1.492) | (1.928) | (2.153) |
| Pseudo R^2^ | 0,414 | 0,417 | 0,420 | 0,417 | 0,421 |
|  |  |  |  |  |  |
| Observations | 355 | 354 | 354 | 354 | 354 |

***Notes***: Robust standard errors in parentheses, clustered for job title. *** p<0.01, ** p<0.05, * p<0.1

**Table C. Questionnaire**

| Items |  |
| --- | --- |
| **Section 1. Socio-demographics** |  |
|  |  |
| **Female:** | (1) Female, (0) Male |
| **Age:** | Number in years |
|  |  |
| **Job role:** | (1) Physicians, (2) Nurses and nurses aides, (3) administrative staff |
|  |  |
| **Children**: *How many children aged 0-5 live in your household?* | Number of children |
|  |  |
| **Dependent adults:** *Are there adults living with you or dependent on you in any way (e.g. art. 104)?* | (1)Yes, (2) No |
| **Housework**: *On average, how many hours per day do you spend on work/care activities outside paid work? (e.g. care and/or education of children, grandchildren; care of elderly/disabled parents; cooking and housework)* | Number of hours |
| **Work-life balance**: *In general, how do your working hours fit in with your family or social commitments outside work?* | (1)Not at all good, (2) Not very good, (3)Moderately good, (4) Good, (5) Very good |
| **BMI:** |  |
| **Height (cms)** | Number in centimetres |
|  |  |
| **Weight (kg)** | Number in Kg |
|  |  |
| **Section 2. Work ability index** |  |
|  |  |
| **Current work ability compared to highest work ability ever:** *Assume that your work ability at its best has a value of 10 points.* *How many points would you give your current work ability*? | (0) Means that you currently cannot work at all; ..; (10) Work ability at its best |
| **Work ability in relation to demands:**   - *How do you rate your current work ability with respect to the* ***physical demands*** *of your work*? - *How do you rate your current work ability with respect to the* ***mental demands*** *of your work*? | (1)Very poor, (2)Rather poor, (3) Moderate, (4)Rather good, (5) Very good |
| **Current diseases:** *In the following list, mark your current diseases diasgnosed by a physician*  *Macro-categories*  01 Injury due to an accident  02 Musculoskeletal disease in back, limbs or other part of the body (e.g. repeated pain in joint muscle, sciatica, rheumatism, arthritis)  03 Cardiovascular disease (e.g. hypertension, coronary heart disease)  04 Respiratory disease (e.g. repeated infections of the respiratory tract, emphysema)  05 Mental disorder (e.g. depression, “burn-out”, anxiety or insomnia)  06 Neurological or sensory disease (e.g. hearing or visual disease, migraine, epilepsy)  07 Digestive disease / condition (e.g. gastritis, gall stones, liver or pancreatic disease, repeated constipation)  08 Genitourinary disease (e.g. infection in urinary tract, gynecological disease or prostate)  09 Skin disease (e.g. allergic or other rash, varicose veins)  10 Tumour or cancer  11 Endocrine or metabolic disease (e.g. diabetes, severe obesity or gout)  12 Blood diseases (e.g. anemia, other blood disorder or defect)  13 Birth defects  14 Other disorder or disease | 1. Yes, physician’s diagnosis (2) No |
| **Estimated work impairment due to diseases***: Is your illness or injury a hindrance to your current job?* | (1) In my opinion I am entirely unable to work  (2) Because of my condition, I feel I am able to do only part time work  (3) I must often slow down my work pace or change my work methods  (4) I must sometimes slow down my work pace or change my work methods  (5) I am able to do my job, but it causes some symptoms  (6) There is no hindrance / I have no diseases. |
| **Illness within last year (12 months):** *During the last 12 months: how many whole days have you been off work because of illness:* | (1) More than 100 days  (2) 25 - 99 days  (3)10 - 24 days  (4) Max. 9 days  (5) None |
| **Estimation of own work ability in 2 years:** *Do you believe, according to your present state of health, that you will be able to do your current job two years from now?* | (1) Unlikely  (2) Not Certain  (3) Relatively certain |
| **Mental capacities**:   - 1. *Considering the last three months: Have you been able to enjoy your regular daily activities?*   2. *Considering the last three months: Have you been active and alert?*   3. *Considering the last three months: Have you felt yourself to be full of hope about the future?* | (0) Never  (1) rather seldom  (2) Sometimes  (3) rather often  (4)Often |
|  |  |
| **Section 3. Working conditions** |  |
|  |  |
| **Shiftwork:** *Do you work shifts?* | (1) Yes, (2) No |
| **Relationship oriented leadership**: *Your immediate boss encourages and supports your development* | (1)Strognly agree; (2) tend to agree; (3) neither agree nor disagree; (4) tend to disagree; (5) strongly disagree |
| **General supervisor support:** *Your immediate boss help and supports you* | (1)Strognly agree; (2) tend to agree; (3) neither agree nor disagree; (4) tend to disagree; (5) strongly disagree |
| **Colleagues support:** *Your colleagues help and support you* | (1)Strognly agree; (2) tend to agree; (3) neither agree nor disagree; (4) tend to disagree; (5) strongly disagree |
| **Autonomy over work goals**: *You are consulted before objectives are set for your work* | (1) Always, (2) Most of the time, (3) sometimes , (4) rarely, (5) never |
| **Autonomy in decision making**: *You can influence decisions that are important for your work* | (1) Always, (2) Most of the time, (3) sometimes , (4) rarely, (5) never |
| **Autonomy in departmental choice:** *You are involved in improving the work organisation or work processes of your department or organisation* | (1) Always, (2) Most of the time, (3) sometimes , (4) rarely, (5) never |
| **Skill match:** *Which of the following statements would best describe your skills in your own work?* | (1)I need further training to cope well with my duties, (2) My present skills correspond well with my duties |

Notes. Socio-demographics (Section 1) were self-developed, the Work Ability Index (Section 2) was constructed using those proposed in Tuomi K., Ilmarinen J., Jankola A., Katajarinne L., Tulkki A. Work Ability Index, Finnish Institute of Occupational Health, while the working conditions (Section 3), was developed inspired by the European Working Conditions Survey (2017).
